# Role of Toll-Like Receptors 2 and 4 in Pulmonary Inflammation and Injury Induced by Pneumolysin in Mice

**DOI:** 10.1371/journal.pone.0007993

**Published:** 2009-11-24

**Authors:** Mark C. Dessing, Robert A. Hirst, Alex F. de Vos, Tom van der Poll

**Affiliations:** 1 Center for Infection and Immunity Amsterdam (CINIMA), Academic Medical Center, University of Amsterdam, Amsterdam, The Netherlands; 2 Center for Experimental and Molecular Medicine, Academic Medical Center, University of Amsterdam, Amsterdam, The Netherlands; 3 Department of Infection, Inflammation and Immunity, University of Leicester, Leicester, United Kingdom; Columbia University, United States of America

## Abstract

**Background:**

Pneumolysin (PLN) is an intracellular toxin of *Streptococcus pneumoniae* that has been implicated as a major virulence factor in infections caused by this pathogen. Conserved bacterial motifs are recognized by the immune system by pattern recognition receptors among which the family of Toll-like receptors (TLRs) prominently features. The primary objective of the present study was to determine the role of TLR2 and TLR4 in lung inflammation induced by intrapulmonary delivery of PLN.

**Methodology/Results:**

First, we confirmed that purified PLN activates cells via TLR4 (not via TLR2) *in vitro*, using human embryonic kidney cells transfected with either TLR2 or TLR4. Intranasal administration of PLN induced an inflammatory response in the pulmonary compartment of mice *in vivo*, as reflected by influx of neutrophils, release of proinflammatory cytokines and chemokines, and a rise in total protein concentrations in bronchoalveolar lavage fluid. These PLN-induced responses were dependent in part, not only on TLR4, but also on TLR2, as indicated by studies using TLR deficient mice.

**Conclusion:**

These data suggest that although purified PLN is recognized by TLR4 *in vitro*, PLN elicits lung inflammation *in vivo* by mechanisms that may involve multiple TLRs.

## Introduction

*Streptococcus pneumoniae* is the most frequently isolated pathogen in community acquired pneumonia [1], [2]. Several pneumococcal proteins and enzymes have been implicated in the virulence of this bacterium and the pathogenesis of pneumonia [3]. Pneumolysin (PLN) is a protein expressed by *Streptococcus pneumoniae* intracellularly and/or in the cell wall, that is present in virtually all clinical isolates [4], [5], [6]. PLN is considered to be an important virulence factor of the pneumococcus. Indeed, mice infected with a PLN-deficient strain of *S. pneumoniae* showed a reduced lethality and a diminished inflammatory response when compared to animals infected with PLN-producing *S. pneumoniae* [7], [8]. PLN remains within the pneumococcus during bacterial growth, but is released when the pathogen autodestructs by expressing autolysin [9] or after destruction by the host immune system or antibiotic treatment [10]. In addition, a recent report raised doubt as to whether PLN truly is an intracellular protein, showing that PLN primarily localized to the cell wall compartment during growth of pneumococci in the absence of detectable cell lysis [6]. At low doses, PLN activates the classical pathway of the complement system, induces cytokine production by macrophages and monocytes, inhibits the migration, respiratory burst and antibacterial activity of neutrophils and macrophages and affects ciliary beating of epithelial cells [11]–[15]. At high doses, PLN can induce cell death; PLN interacts with cholesterol in the host-cell membrane resulting in the formation of transmembrane pores and death of the host (immune) cell [16]. Our laboratory recently demonstrated that purified PLN induces neutrophil influx and the production of cytokines and chemokines in the lungs of mice [17]. In addition, PLN dose dependently induced vascular permeability and pulmonary edema in mice [18], [19]. Together these data suggest that PLN has a strong impact on the host response to invasion of the lower respiratory tract by *S. pneumoniae*.

Toll-like receptors (TLRs) are pattern recognition receptors that sense the presence of microorganisms by virtue of their capacity to recognize pathogen associated molecular patterns [20]. Recent studies have shown that PLN is recognized by TLR4 *in vitro* [21]–[25]. Knowledge on the role of TLRs in PLN-induced inflammation in the lung *in vivo* is limited: one investigation reported that the induction of plasminogen activator inhibitor type (PAI-1) I gene expression by PLN in the lungs of mice at least in part depends on TLR4 [26]. The primary objective of this study was to determine the roles of TLR2 and TLR4 in the pulmonary effects of purified PLN in mice *in vivo*.

## Methods

### Cell Cultures

Human embryonic kidney (HEK)-293 cells [27] transfected with CD14 and TLR2 or TLR4 (kindly provided by Douglas Golenbock, Division of Infectious Diseases and Immunology, University of Massachusetts Medical School, Worcester, MA) were grown in DMEM (1 mM pyruvate, 2 mM L-glutamine, penicillin, streptomycin and 10% fetal bovine serum). The murine alveolar macrophage cell line MH-S (American Type Culture Collection, Rockville, MD) was grown in RPMI 1640 (1 mM pyruvate, 2 mM L-glutamine, penicillin, streptomycin and 10% fetal bovine serum). *In vitro* stimulation was conducted in 96-well plates (Greiner, Alphen aan de Rijn, the Netherlands) at a density of 1×10^5^ cells/ml. All cell lines were allowed to adhere overnight at 37°C in a humidified atmosphere containing 5% CO_2_ and stimulated the next day for 6 hours. For all stimulations, HEK cells were co incubated with supernatant from MD-2-excreting HEK cells [27]–[29] together with either highly purified PLN (purified from *Escherichia coli* strain JM109 expressing a functional *pln* gene as previously described [30]), lipopolysaccharide (LPS from *Escherichia coli* O111:B4, Sigma Aldrich, St. Louis, MO) or lipoteichoic acid (LTA from *Staphylococcus aureus*) [31]. Contamination of LPS in our PLN preparation was 4.6 ng/mg PLN as determined with the chromogenic Limulus Amoebocyte Lysate assay (LAL assay) [32]. In some experiments polymyxin B (Sigma Aldrich) was used at 10 µg/ml.

### MTT Assay

Supernatant of stimulated cells was removed and cells were incubated for 1–2 hours at 37°C with 100 µl 10% MTT (3-(4,5-dimethylthiazol-2-yl)-2,5-diphenyltetrazolium bromide, Sigma Aldrich) solution (5 mg/ml) in medium. Thereafter MTT solution was replaced and cells were incubated with acetic isopropanol and firmly resuspended to dissolve violet crystals and incubated for 10 minutes. OD of 560 nm was used to measure metabolic activity and corrected for cell debris by OD 655 nm [33].

### Animals

Specific pathogen free 8–10 weeks old C57BL/6 wild-type (WT) mice were purchased from Charles River (Maastricht, The Netherlands). TLR2 knockout (KO) mice and TLR4 KO mice (kindly provided by Shizuo Akira, Exploratory Research for Advanced Technology, Japan Science and Technology Agency, Suita, Osaka, Japan) were generated as described previously [34], [35] and backcrossed six times to a C57BL/6 background. All mice were bred in the animal facility of the Academic Medical Center in Amsterdam. Age and sex matched mice were used in all experiments. All experiments were approved by the Animal Care and Use Committee of the University of Amsterdam.

### *In Vivo* Stimulation

Intranasal inoculation of PLN was performed as described earlier [17]. Briefly, mice were lightly anesthetized by inhalation of isoflurane (Upjohn, Ede, the Netherlands) after which 50 µl of sterile phosphate-buffered saline (PBS) or PLN dissolved in PBS was administered intranasally. After 6 hours, mice were sacrificed and bronchoalveolar lavage (BAL) was performed. For this the trachea was exposed through a midline incision and cannulated with a 22-gauge Abbocath-T catheter (Abbott, Sligo, Ireland). The lavage was performed by instilling two 0.5-ml aliquots of PBS. Lavage fluid was retrieved thereafter and counted using Z2 Coulter particle count and size analyzer (Beckman-Coulter Inc., Miami, FL.). Differential cell counts were determined in BAL fluid (BALF) using cytospin preparations stained with modified Giemsa stain (Diff-Quick; Baxter, McGraw Park, Ill).

### Assays

Mouse tumor necrosis factor (TNF)-α, interleukin (IL)-1β, IL-6, cytokine-induced neutrophil chemoattractant (KC) and macrophages inflammatory protein (MIP)-2 and human IL-8 were measured by species specific ELISA's (R&D Systems, Minneapolis, MN). Total protein level was measured by BCA protein assay (Pierce, Rockford, IL).

### Statistical Analysis

Data are expressed as means±SEM or median+interquartile range. Differences were analyzed by Student T-test (*in vitro* cell stimulation) or Mann Whitney U test (***in vivo*** **stimulation**). A value of P<0.05 was considered statistically significant.

## Results

### PLN Is Recognized by TLR4 *In Vitro*

**Figure 1:**
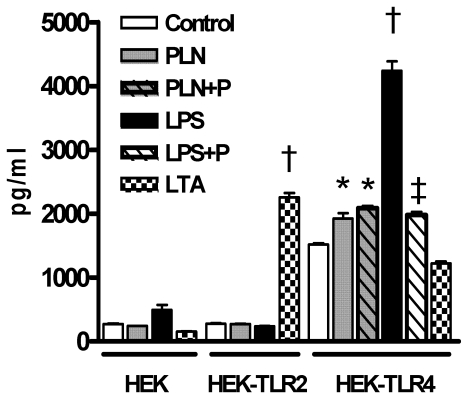
PLN activates HEK cells via TLR4. IL-8 production in HEK-293 cells transfected with CD14 and either TLR2 or TLR4 were incubated with medium (control), LPS (100 ng/ml), LTA (5 µg/ml) or PLN (1 µg/ml) for 6 hours. In some experiments polymyxin B (P) was used at 10 µg/ml. Data are mean±SEM (N = 4 per group). * P<0.01 versus control, † P<0.001 versus control, ‡P<0.001 versus LPS.

Earlier studies have shown that PLN is recognized by TLR4 [21]–[25]. To confirm that our PLN preparation is recognized by TLR4, we incubated HEK cells transfected with either TLR2 or TLR4, with PLN. As positive controls for TLR4 and TLR2 signaling we also incubated HEK cells with LPS or LTA respectively (Figure 1). HEK cells not transfected with TLR2 or TLR4 did not respond to PLN, LPS or LTA. As expected, LTA and LPS – induced IL-8 production was dependent on the presence of TLR2 and TLR4 respectively (both P<0.001 versus medium control). PLN elicited IL-8 release by HEK-TLR4 (P<0.05 versus medium control) but not by HEK-TLR2 cells. Polymyxin B significantly inhibited LPS-TLR4 signaling but did not influence the effect of PLN on HEK-TLR4 cells, indicating that contaminating LPS can not explain the capacity of PLN to stimulate TLR4. Of note, HEK-TLR4 cells spontaneously produced more IL-8 compared to the other two HEK cell lines. Over expression of TLR4 in HEK cells has been shown to constitutively activate NF-κB resulting in a spontaneous activated condition [36] which could explain the elevated spontaneous release of IL-8. Overall, these data confirm earlier studies [21]–[25], showing that PLN is recognized by TLR4.

### Dose-Dependent Inflammatory and Lytic Properties of PLN *In Vitro*

**Figure 2:**
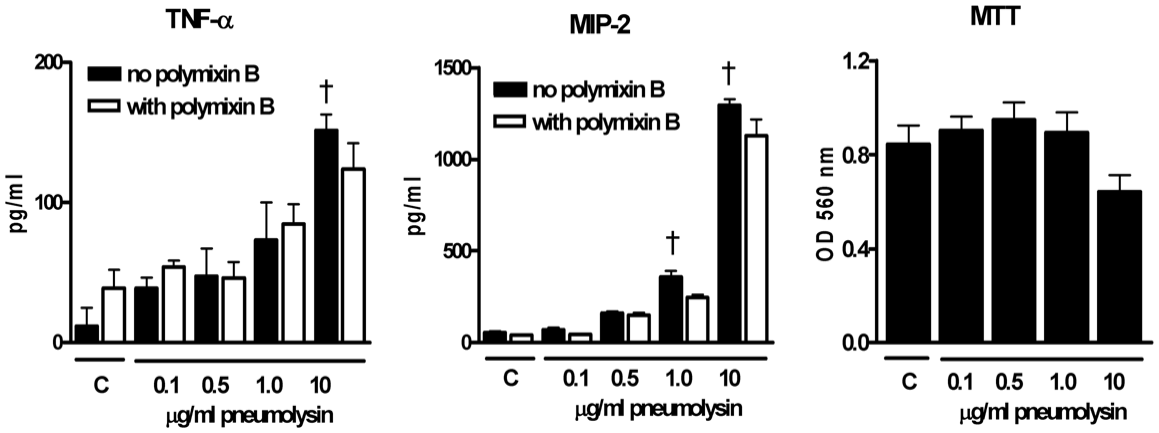
Inflammatory and cytolytic effects of PLN on mouse alveolar macrophage MH-S cells. MH-S cells were incubated with increasing doses of PLN for 6 hours with/without polymyxin B (10 µg/ml ) and TNF-α, MIP-2 and cell death were determined thereafter. Cell death was measured using MTT assay as described in Methods section. Data are mean±SEM (N = 5 per group). * P<0.05, † P<0.01 versus control.

Alveolar macrophages in lungs can interact with PLN after intranasal inoculation. To investigate PLN-induced cytokine production and lysis of cells, we incubated mouse alveolar macrophage MH-S cells with increasing doses of PLN for 6 hours (corresponding with the observation period used in our *in vivo* experiments – see further). TNF-α and MIP-2 production from MH-S cells (Figure 2) increased dose dependently after incubation with PLN. PLN-induced TNF-α and MIP-2 production was not affected by the LPS inhibitor polymyxin B. PLN is known to induce lysis of cells when incubated at high doses by inducing pores into the cell membrane [16]. To further investigate the lytic properties of PLN we determined cell metabolic activities by MTT assay; a tool to measure the induction of cell death [33]. Overall cell metabolic activity was reduced in MH-S cells incubated with the highest PLN dose (10 µg/ml), indicative of enhanced cell death (Figure 2; P = 0.06 compared to medium control). These data suggest that PLN activates alveolar macrophages to produce cytokines and/or chemokines at low doses, whereas higher doses cause cell death.

### Role of TLR2 and 4 in PLN-Induced Lung Inflammation and Injury *In Vivo*

**Figure 3:**
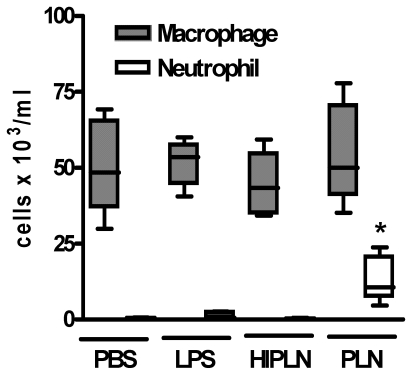
Cell composition in BALF of PBS-, LPS-, PLN- and heat inactivated PLN-treated mice. Macrophage and neutrophil counts in BALF from WT mice, 6 hours after inoculation of PBS, LPS (2 pg/mouse), heat inactivated PLN (HIPLN 500 ng/mouse) or PLN (500 ng/mouse). Data are plotted in Box&Whiskers graph (median+interquartile range N = 5 per group). * P<0.05 versus PBS.

**Figure 4:**
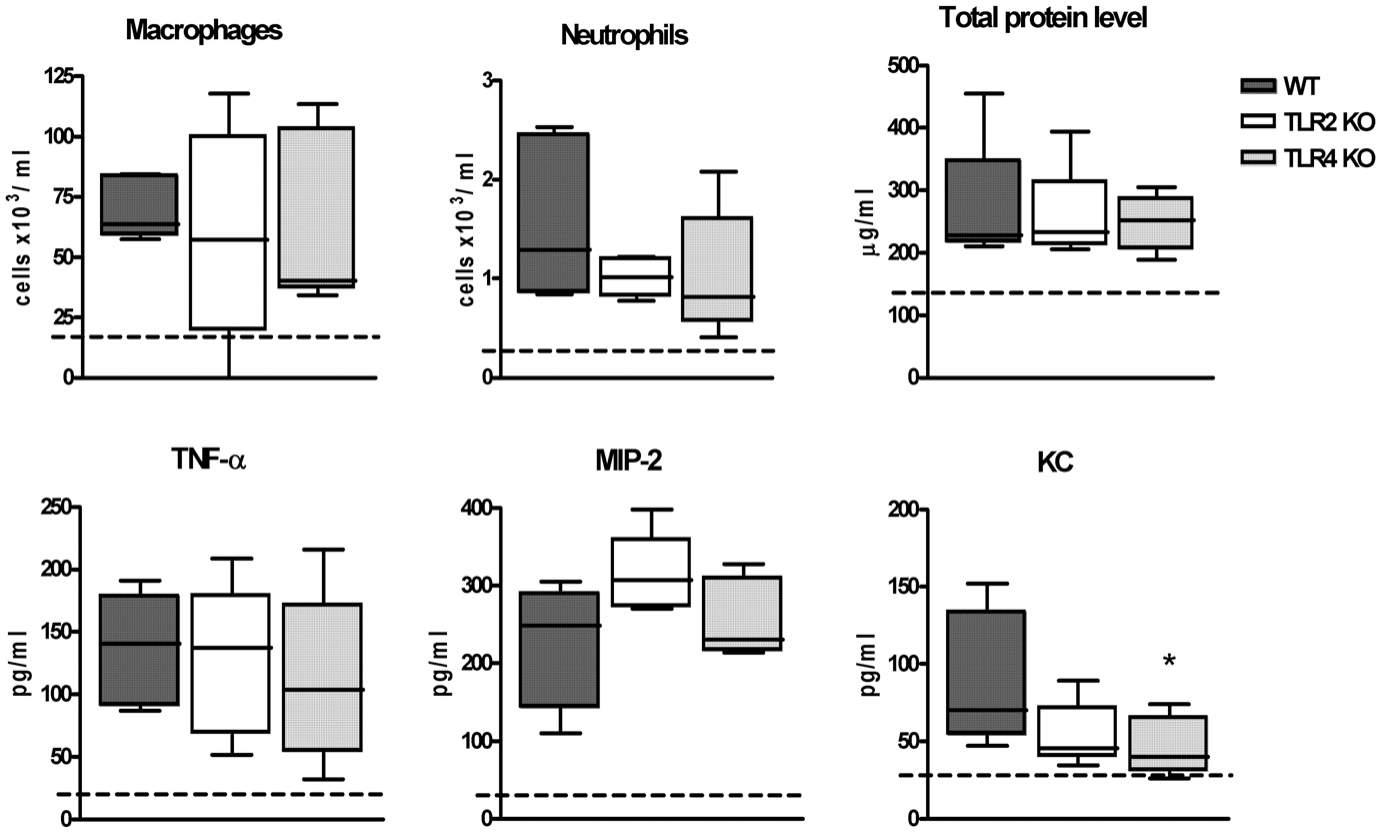
Roles of TLR2, and TLR4 in lung inflammatory response to low dose PLN *in vivo*. Macrophage and neutrophil counts, total protein and TNF-α, MIP-2 and KC concentrations in BALF from WT, TLR2 KO and TLR4 KO mice, 6 hours after inoculation of 25 ng/mouse Data are plotted in Box&Whiskers graph (median+interquartile range N = 8 per group) * P<0.05 versus WT mice. Dotted line indicates mean value of PBS-treated mice.

**Figure 5:**
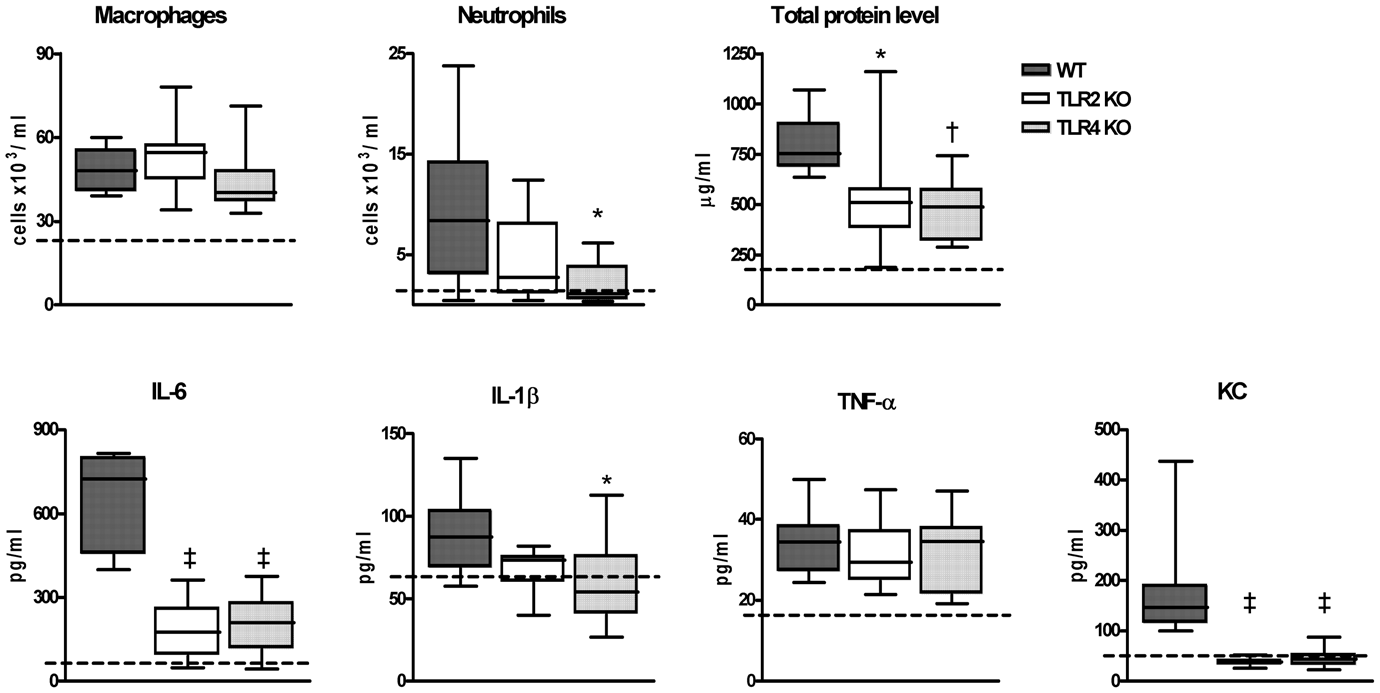
Roles of TLR2 and TLR4 in lung inflammatory response to high dose PLN *in vivo*. Macrophage and neutrophil counts, total protein, IL-6, IL-1β, TNF-α and KC concentrations in BALF from WT, TLR2 KO and TLR4 KO mice, 6 hours after inoculation of 500 ng/mouse. Data are plotted in Box&Whiskers graph (median+interquartile range N = 8 per group). * P<0.05, † P<0.01, ‡ P<0.001 versus WT mice. Dotted line indicates mean value of PBS-treated mice.

Previous studies have documented the capacity of PLN to induce lung inflammation and injury in rodents *in vivo* [17], [18], [37]. In preliminary experiments we first confirmed that PLN causes dose-dependent effects in the lungs of WT mice upon intranasal administration with respect to recruitment of neutrophils and release of cytokines and chemokines into the bronchoalveolar space, and with regard to pulmonary vascular leakage as determined by total protein levels in BALF (see below and data not shown). To exclude biased effects due to contaminating LPS in our PLN preparation, we performed two control experiments. First, we inoculated WT mice with the amount of LPS present in our highest dose (500 ng PLN/mouse; see further) which, according to the LAL assay, was 2 pg LPS/mouse. In addition, we inoculated WT mice with 500 ng/mouse heat inactivated PLN (80°C, 60 minutes), considering that LPS is heat stable. After 6 hours mice were sacrificed and differential cell counts were determined in BALF. Mice treated with either 2 pg LPS or 500 ng heat inactivated PLN showed a similar cellular composition of BALF when compared with PBS treated mice, which was in contrast to the neutrophil influx induced by PLN (Figure 3). These data indicate that the effects produced by PLN can not be explained by contaminating LPS. Thereafter we investigated the roles of TLR2 and TLR4 in the effects of two PLN doses: one dose that caused modest lung inflammation and vascular leakage (25 ng/mouse, Figure 4) and one that caused profound lung inflammation and injury (500 ng/mouse, Figure 5). Intranasal administration of PLN at a dose of 25 ng induced a modest influx of leukocytes into BALF, which was caused by an increase in the number of alveolar macrophages and neutrophils (P<0.05 versus PBS controls). In addition, PLN induced increases in the BALF levels of TNF-α, MIP-2 and KC (all P<0.05 versus PBS controls), whereas IL-6 and IL-1β concentrations remained low and similar to PBS control mice (data not shown). Moreover, PLN 25 ng elicited a modest rise in BALF total protein concentrations (P<0.05 versus PBS controls). These pulmonary responses to low dose PLN were unaltered in TLR2 and TLR4 KO mice with the exception of KC levels in BALF of TLR4 KO mice, which were reduced (P<0.05 compared to WT mice).

Having established that the contribution of TLR2 and TLR4 to the lung inflammatory response to low dose PLN was negligible, we next inoculated mice with a higher dose of PLN (500 ng/mouse). At this dose PLN elicited macrophage and neutrophil influx, release of IL-6, IL-1β, TNF-α and KC and a rise in total protein level in BALF of WT mice (all P<0.05 versus PBS control). MIP-2 levels remained low and similar to PBS control mice (data not shown). Six hours after intranasal administration of 500 ng PLN, TLR4 KO mice displayed reduced neutrophil influx, diminished IL-6, IL-1β and KC release and lower total protein levels in BALF when compared with WT mice (P<0.05 to P<0.001). Surprisingly, TLR2 KO mice also demonstrated significantly reduced BALF levels of IL-6, KC and total protein compared to WT mice (P<0.05). These observations were confirmed in a second independent experiment (data not shown). BALF TNF-α levels were similar in WT, TLR2 KO and TLR4 KO mice. Twenty-four hours after inoculation of PLN at 500 ng/mouse, the BALF cell composition was similar in WT, TLR2 KO and TLR4 KO mice and cytokine and chemokine levels were undetectable in all three mouse strains (data not shown). These data suggested that the induction of lung inflammation and injury by high dose PLN was dependent on the presence of TLR2 and TLR4.

## Discussion

The pneumococcal cell wall consists of several proteins and enzymes that contribute to the virulence of the pathogen and the pathogenesis of pneumonia [3]. PLN is a toxin of *S. pneumonia*, expressed intracellularly and/or in the cell wall compartment, that is present in all clinical isolates [4], [5], [6]. Several studies have demonstrated that PLN is recognized by the immune system through a specific interaction with TLR4 [21]–[25]. The primary objective of the present investigation was to determine the contribution of TLR2 and TLR4 in lung inflammation and injury induced by PLN *in vivo*. First we confirmed that our PLN preparation activated HEK cells via TLR4. We then revealed that intrapulmonary delivery of PLN induces an inflammatory response in the mouse lung that is dependent in part not only on TLR4, but also on TLR2. Our data expand a recent investigation showing that the induction of PAI-1 mRNA in the lungs of mice at least in part depends on TLR4 [26].

Several *in vitro* studies have shown that low doses of PLN induce proinflammatory reactions in immune cells like neutrophils, macrophages, monocytes, dendritic cells and epithelial cells (10, 13, 22, 24, 37, 38). Alveolar macrophages interact with respiratory pathogens upon invasion of the lower airways. Alveolar macrophages responded to PLN by production of cytokines and/or chemokines in a dose dependent manner, whereas high PLN doses caused cell death. Our current findings of PLN-induced lung inflammation in WT mice confirm and extend previous studies. Several investigations reported lung injury and leakage of the alveolar-endothelial barrier resulting in pulmonary edema after pulmonary instillation and aerosol delivery of PLN [19], [38]. In addition, installation of PLN resulted in depletion of the alveolar macrophage pool and influx of neutrophils and monocytes; PLN-induced lung injury was associated with only a small increase in TNF-α and MIP-2 levels in BALF [38]. In a study performed earlier in our laboratory, intranasal installation of PLN dose dependently induced neutrophil influx and IL-6, KC and MIP-2 production in the bronchoalveolar compartment [17]. Here we utilized this model of PLN-induced lung inflammation to determine the contribution of TLR2 and TLR4 to PLN effects *in vivo*. In line with previous *in vitro* studies [21]–[25], PLN responses in the lungs were (in part) TLR4 dependent: in particular KC release relied on the presence of TLR4, whereas other responses (neutrophil influx, protein leakage, cytokine release) were significantly reduced in TLR4 KO mice only after administration of high dose PLN. These latter findings are in accordance with a recent study that reported attenuated induction of PAI-1 mRNA in lungs of TLR4 KO mice upon intrapulmonary delivery of high dose PLN [26]. Remarkably, in the current investigation also TLR2 KO mice displayed a reduced responsiveness to PLN and this attenuated phenotype was not much different from that of TLR4 KO mice. A possible explanation could be that PLN induces endogenous ligands which may signal through TLR2 (and/or TLR4) [39], [40]. One of these danger associated ligands is hyaluronan [40]. However, BALF hyaluronan levels were even lower in TLR2 KO and TLR4 KO mice than in WT mice (data not shown), suggesting that hyaluronan concentrations in BALF may at least in part reflect pulmonary leakage. This however does not exclude that PLN induces other endogenous ligands which could signal through TLR2 and/or TLR4. The concept of endogenous TLR ligands amplifying host responses to inflammatory triggers is supported by our recent findings that highly purified LTA, which is an established TLR2 ligand [32], [41]–[44], induces less profound lung inflammation not only in TLR2 KO mice, but also in TLR4 KO mice [45] 47). The only earlier study that examined the contribution of TLRs in PLN-induced pulmonary effects *in vivo* did not address the role of TLR2 [26]. In our experiments we could not confirm a role for endogenous TLR2 or TLR4 expressed by alveolar macrophages in PLN-induced KC or TNF-α release, since primary alveolar macrophages did not respond to PLN in doses up to 1 µg/mL (data not shown). It should be noted that PLN can induce inflammation by mechanisms that do not rely on TLRs. In particular, PLN exerts complement-activating activity that is mediated by a part of the toxin that is different from that mediating cytotoxic activity, and mutant pneumococci in which either the cytotoxic or complement-activating activity of PLN was deleted both showed a reduced virulence in mice [46].

Surprisingly, the lower PLN dose induced higher cytokine levels in BALF of mice than the higher PLN dose, which was especially true for TNF-α. Although we do not have a firm explanation for this, differences in viability of cytokine-producing cells within the lungs and differences in the kinetics of cytokine release into the bronchoalveolar space upon administration of increasing PLN doses may have played a role.

The PLN used in this study was manufactured according to the method of Mitchell et al. which results in highly purified pneumococcal PLN [30]. Several experiments were done to exclude that PLN-induced effects were caused by LPS contamination. First, polymyxin B, an established LPS inhibitor, did not influence PLN effects on HEK or MH-S cells. Secondly, the highest PLN concentration used *in vivo* contained 2 pg LPS/mouse which did not induce an immune response in WT mice. In addition, heat inactivated PLN did not induce lung inflammation in WT mice *in vivo*, which - considering that LPS is heat stabile - further argues against LPS contamination. Moreover, the addition of soluble MD2 to HEK-TLR4 cells was absolutely required for LPS responsiveness, confirming previous reports [27]–[29], whereas PLN induced similar cytokine release by these cells in the absence or presence of soluble MD2 (data not shown). Finally, the fact that HEK-TLR2 cells did not respond to PLN argues against contaminating TLR2 ligands.

PLN is a major virulence factor in *S. pneumoniae* infections. We here show that PLN induces inflammation in the bronchoalveolar compartment of mice via mechanisms that rely in part on TLR2 and TLR4. Investigations seeking to unravel the complex interactions between pneumococcal components and host immune cells in the lung may assist in understanding pathophysiological mechanisms at play during pneumonia caused by *S. pneumoniae*.
